# Therapeutic Potential of Targeting Malt1-Dependent TCR Downstream Signaling to Promote the Survival of MHC-Mismatched Allografts

**DOI:** 10.3389/fimmu.2020.576651

**Published:** 2020-09-11

**Authors:** Lerisa Govender, Josip Mikulic, Jean-Christophe Wyss, Olivier Gaide, Margot Thome, Dela Golshayan

**Affiliations:** ^1^Transplantation Centre and Transplantation Immunopathology Laboratory, Department of Medicine and Service of Immunology, Lausanne University Hospital (CHUV) and University of Lausanne (UNIL), Lausanne, Switzerland; ^2^Department of Medicine and Service of Dermatology, Lausanne University Hospital (CHUV) and University of Lausanne (UNIL), Lausanne, Switzerland; ^3^Department of Biochemistry, University of Lausanne (UNIL), Epalinges, Switzerland

**Keywords:** transplantation, paracaspase, NF-kB, calcineurin inhibitors, regulatory T cells, Th17 cells

## Abstract

Strategies targeting T cells are the cornerstone of immunosuppression after solid organ transplantation. The transcription factor NF-κB is a key regulator of downstream T-cell activation and induction of inflammatory mediators; its full activation via antigen receptor engagement requires both the scaffold and the protease activity of the paracaspase Malt1. Experimental studies have highlighted that Malt1-deficient mice were resistant to experimental autoimmune encephalomyelitis, although they lacked peripheral regulatory T cells (Treg). Here, we compared targeting Malt1 versus using calcineurin inhibitors as immunosuppression in a stringent experimental transplantation model. We found that Malt1-deficiency impaired Th1-mediated alloresponses *in vitro* and *in vivo* and significantly prolonged MHC-mismatched skin allograft survival, compared to cyclosporine. However, it paradoxically enhanced Th17 differentiation in the transplantation setting. Interestingly, more selective inhibition of Malt1 protease activity in wild-type mouse and human peripheral T cells *in vitro* led to attenuation of alloreactive Th1 cells, while preserving preexisting Treg in the peripheral T-cell pool, and without promoting Th17 differentiation. Thus, there is a place for further investigation of the role of Malt1 signaling in the setting of transplantation.

## Introduction

CD4^+^ T cells play a central role in primary alloresponses after solid organ transplantation (SOT) by providing effector cytokines and cognate help for cytotoxic CD8^+^ T lymphocytes and B-cell activation. Graft rejection has been shown to be primarily a Th1-mediated immune response; however, Th17- or Th2-mediated graft rejection has been observed, in particular in the absence of a robust Th1 response (1–3). T-cell activation is regulated by a set of transcription factors belonging to the nuclear factor kappa-B (NF-κB) and nuclear factor of activated T cells (NFAT) families (4). The ligation of the T-cell receptor (TCR) by cognate antigens leads to increased levels of intracellular calcium and activation of the calcium-dependent phosphatase enzyme calcineurin that dephosphorylates NFAT. NFAT then translocates into the nucleus and binds to the enhancer of the gene encoding interleukin-2 (IL-2). By blocking this downstream TCR signaling, calcineurin inhibitors (CNI) prevent T-cell activation and downstream transcription of *IL-2*. Despite the success of CNI in preventing acute rejection they cannot fully control chronic immune activation leading to graft dysfunction (5). Moreover, the use of CNI is associated with side effects including cardiovascular and renal toxicities contributing to patient’s morbidity (3). Alternatively, inhibition of the classical NF-κB signaling pathway could be considered as an immunosuppressive strategy.

NF-κB signal transduction is a key event in antigen-dependent lymphocyte activation. In resting cells, NF-κB is sequestered in the cytoplasm. TCR/BCR antigen engagement results in the assembly of the caspase-recruitment domain containing membrane-associated guanylate kinase 1 (CARMA1, also known as CARD11), together with B-cell lymphoma 10 (Bcl-10) and mucosa-associated lymphoid tissue lymphoma translocation gene 1 (Malt1) into the CBM complex (6, 7). Malt1 is essential for NF-κB-dependent lymphocyte activation, since Malt1-deficient (Malt1-ko) mice have defects in the activation and proliferation of T and B cells, and suffer from general immunodeficiency (8, 9). Malt1 can promote NF-κB activation as a scaffold protein, by recruiting the ubiquitin ligase tumor necrosis factor receptor-associated factor 6 (TRAF6) that activates the inhibitor of κB (IκB) kinase (IKK) complex. IKK-mediated phosphorylation of the NF-κB inhibitor, IκB, promotes its proteasomal degradation. This allows NF-κB to enter the nucleus and bind specific DNA sequences that control the transcription of genes encoding key molecules involved in inflammation, cell survival and division, including IL-2. Malt1 also promotes T-cell activation and differentiation via its proteolytic activity, by cleaving proteins such as A20 and RelB that regulate NF-κB activation independently of IκB, and by cleaving proteins such as Regnase-1 and Roquin that modulate transcript stability (10–13).

Experimental models have reported up-regulation of NF-κB activity in cardiac allografts at several time-points after transplantation compared to control tissues (14, 15). Mice with either CARMA1-deficiency or impaired T-cell specific NF-κB signaling had reduced ability to reject allografts (16–18). Interestingly, the induction of long-term allograft survival in these mice was dependent on the type of transplanted tissue. Indeed, whilst the impairment of NF-κB signaling resulted in prolonged survival of heart allografts, it was not sufficient to promote tolerance toward more immunogenic skin allografts. Available immunosuppressive agents such as steroids or proteasome inhibitors target NF-κB, but these drugs are not cell-specific and could be associated with systemic side-effects (19).

Here, we investigated whether more selective impairment of NF-κB signaling, by specifically targeting Malt1, would prove effective in promoting allograft survival. We report that Malt1-deficiency attenuated Th1-mediated alloresponses *in vitro* and *in vivo*, resulting in significantly prolonged MHC-mismatched skin graft survival, even with very low frequencies of peripheral CD4^+^Foxp3^+^ regulatory T cells (Treg). Additionally, Malt1-deficieny induced tolerance to minor histocompatibility antigens (minor-H)-mismatched skin grafts. Moreover, pharmacological inhibition of Malt1 protease activity in wild-type mouse and human T cells *in vitro*, regulated alloresponses without affecting preexisting Treg. We therefore propose that in the setting of SOT, targeting Malt1 could be an effective strategy to dampen immune responses against MHC-mismatched allografts.

## Materials and Methods

### Mice

Wild type (Wt) C57BL/6N (B6, H2^*b*^) and B6xDBA2 F1 (B6D2, H2^*b*^xH2^*d*^) mice were purchased from Charles River and Janvier Laboratories, France. Malt1-deficient mice (Malt1-ko, C57BL/6N) were kindly provided by Vishva Dixit (Genentech, San Francisco, CA, United States) (8). Malt1-knock-in (Malt1-ki, C57BL/6-Malt1tmC472A) mice were described (20). Experimental procedures were performed on 6–12 weeks old mice, in accordance with Canton de Vaud veterinary authorizations (N° 2655.0), in specific pathogen-free animal facilities.

### Skin Transplantation

Full-thickness B6D2 or male B6 tail skins were grafted on beds prepared on lateral flanks of sex-matched or female B6 recipients. Graft sites were protected under sterile gauze covered by a plaster, removed at day 10. Grafts were observed daily afterward and considered rejected when no viable skin remained. For immunosuppression, mice were injected intraperitoneally (i.p.) with 10 mg/kg cyclosporine (CsA; Sandimmun^®^, Novartis Pharma) once a day post-transplantation, or received mepazine (Calbiochem) i.p. daily at 15 mg/kg, starting the day before transplantation (21). All the *in vivo* experiments were repeated at least 3 times.

### Purification of Mouse T Cells

CD4^+^ T cells were negatively selected from mouse spleens and lymph nodes single-cell suspensions after incubation with the following rat anti-mouse hybridoma culture supernatants: anti-MHC class II (M5/114, TIB-120/ATCC; Manassas, VA, United States), anti-CD45R/B220 (RA3-3A1, TIB-146/ATCC), anti-CD16/32 (2.4G2, HB-197/ATCC), anti-CD8 (YTS169; Therapeutic Immunology Group, Oxford, United Kingdom), followed by sheep anti-rat DynaBeads^®^ (Invitrogen) before separation in a magnetic field. CD4*^+^*CD25^–^ and CD4^+^CD25^+^ subsets were selected using anti-CD25-biotinylated (clone 7D4, BD Biosciences) monoclonal antibody (mAb) followed by Streptavidin-MicroBeads^®^ (MiltenyiBiotec, Germany) and separation on a magnetic column (22).

### Generation of Mouse Dendritic Cells

Fresh bone marrow (BM) cells flushed from femurs and tibias were passed through 70-μm cell-strainers. After erythrocytes lysis, BM cells were incubated with supernatants from YTS169, YTS191, M5/114, RA3-3A1 followed by sheep anti-rat Dynabeads^®^. After magnetic separation, cells were transferred to 24-well plates in RPMI-10% FCS with 20 ng/ml mouse recombinant granulocyte/macrophage colony-stimulating factor (GM-CSF, R&D Systems), with change of medium and removal of small non-adherent cells on days 2 and 4. For maturation, 1 μg/ml LPS (*E. coli* 026:B6, Sigma) was added on day 6 for the final 12 h.

### Mouse Cell Cultures

All cultures were performed in RPMI-1640 (Sigma) supplemented with 100 IU/ml penicillin, 100 μg/ml streptomycin, 2 mM L-glutamine, 0.01 M Hepes, 50 μM 2β-mercaptoethanol (Invitrogen) and 10% heat-inactivated fetal calf serum (FCS) (EuroClone, United Kingdom). Purified mouse T cells were stimulated with anti-CD3/CD28-coated beads (Dynabeads^®^ Mouse T-Activator) at 1:1 bead-per-cell ratio for 3 days. Alternatively, mixed leucocytes reactions (MLR) were performed for 5 days, with 1:5 ratio of allogeneic dendritic cells (DC) to T cells. T-cell proliferation was measured by CFSE labeling (5 μM; Molecular probes, NL) and flow cytometry analysis of CFSE dilutions. Wt T-cell proliferation was also assessed in the MLR in the presence of the Malt1 tetrapeptide inhibitor z-VRPR-fmk [Bachem, Switzerland (23)] or cyclosporine A (CsA; Sandimmun^®^, Novartis Pharma). All culture conditions were performed in triplicate wells.

### Antibodies and Flow Cytometry

The following anti-mouse fluorochrome-conjugated mAbs and respective isotype controls were used: CD4 (clone RM4-5), CD8 (53-6.7), CD44 (IM7), CD62L (MEL-14), IL-2 (JES6-5H4), IFN-γ (XMG1.2), IL-17 (TC11-18H10), CD25 (PC61) and Foxp3 (FJK-16s) purchased from BD Biosciences and eBioscience. For intracellular cytokines analysis, cells were re-suspended at 2 × 10^6^ cells/mL in RPMI-10% FCS, re-stimulated with 50 ng/mL phorbol myristate acetate (PMA), 0.5 μg/mL ionomycin for 5 h and 10 μg/mL Brefeldin A (Sigma). Cells were then harvested, surface stained, fixed for 10′ in BD Facs Lysing Solution, washed and permeabilized (Permeabilization Buffer, Becton Dickinson AG) before intracellular staining. For Foxp3, we used the Foxp3 staining kit (eBioscience). For the analysis of p-Stat3 and p-Stat5 expression, T cells were stimulated using anti-CD3/CD28-coated beads for 20′ at 37°C. Cells were then washed and permeabilized with pre-chilled Perm Buffer III (BD Biosciences) for 30′ on ice, re-suspended in FACS buffer and stained with BD Phosflow^TM^ mAbs for 60′ at room temperature. Flow cytometry acquisition was done on FACS-Calibur^TM^ using CellQuest^TM^ and analyzed using Flow Jo software.

### Isolation of Human Peripheral Blood Mononuclear Cells

Blood samples were collected from healthy donors by the regional transfusion center to prepare individual buffy coats. Peripheral blood mononuclear cells (PBMC) were isolated from blood by using Ficoll-Paque Plus (GE Healthcare) and density gradient centrifugation. Antigen-presenting cells (APC) were enriched from total PBMC, as previously reported (24).

### Human Cell Cultures

APC were stimulated overnight using LPS (10 ng/ml) in complete RPMI 1640 medium supplemented with 10% of fetal bovine serum (FBS heat-inactivated, Euroclone), 0.01 M Hepes and non-essential amino acids (NEAA 1X, GIBCO), 100 IU/ml penicillin and 100 μg/ml streptomycin, 2 mM glutamate and pyruvate. PBMC were co-incubated with enriched irradiated (30 Gy) APC at a 1:2 responder/stimulator cells ratio, alone or with the addition of different concentrations of mepazine hydrochloride (CAS 738596-90-2, Calbiochem) a Malt1 inhibitor II, for 7 days at 37°C.

### Flow Cytometry Analysis of Human Cells

At day 7 of co-culture, PBMC were harvested and briefly re-stimulated with 50 ng/mL PMA, 0.5 μg/mL ionomycin and 10 μg/mL Brefeldin A, for 4 h at 37°C for intracellular cytokines detection. Dead cells were identified using the LIVE/DEAD^TM^ Fixable Dead Cells kit (Invitrogen) following the manufacturer’s instructions. The following anti-human fluorochrome-conjugated Abs were used for surface and intracellular labeling: CD3 (clone SK7), CD4 (SK3), CD25 (M-A251), CD127 (eBioRDR5), Foxp-3 (PCH101), Ki-67 (KI-67), IL-2 (MQ1-17H12), IL-17 (BL168), IFNγ (B27). Abs and corresponding isotype controls were purchased from BD Biosciences, BioLegend and eBioscience. Flow cytometry acquisition was done on a Gallios machine (Beckman Coulter) and analyzed using Flow Jo software.

### Statistical Analysis

Unpaired two-tailed Students *t*-test and one-way ANOVA multiple comparison tests (GraphPad Prism version 6 software) were used to calculate significance levels between experimental groups. Median graft survival times (MST) between groups were analyzed using Kaplan-Meier curves and the log-rank test. *P* values <0.05 were considered significant.

## Results

### Malt1-Deficiency Prolongs Survival of MHC-Mismatched Skin Allografts by Dampening the Production of Pro-inflammatory Th1 Cytokines

**FIGURE 1:**
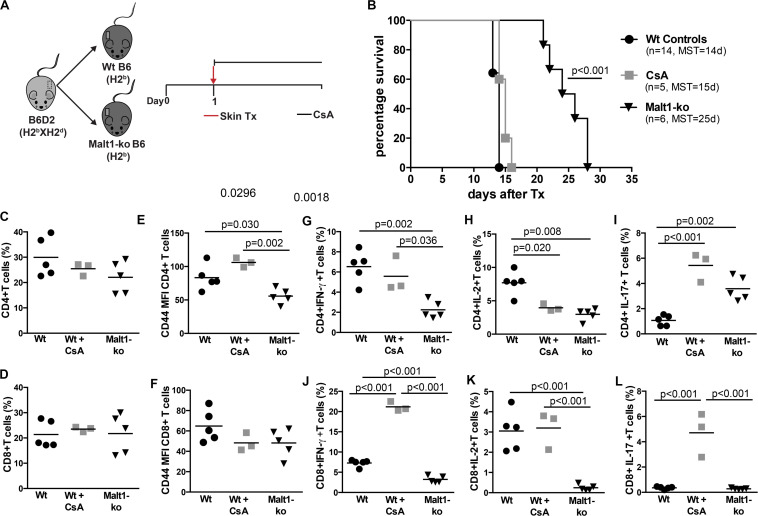
Malt1-deficiency prolongs MHC-mismatched skin graft survival. **(A)** To evaluate the role of TCR signaling mediated via Malt1 in alloresponses, recipient Malt1-ko and wild-type (Wt) B6 mice received a MHC-mismatched B6D2 skin allograft. As a comparison group, Wt mice were treated with cyclosporine A (CsA) i.p. daily after transplantation. **(B)** Graft survival. Graft draining lymph nodes (dLN) were harvested at rejection and analyzed by flow cytometry. **(C,D)** Frequency of CD4^+^ and CD8^+^ T cells, respectively. **(E,F)** Mean fluorescence intensity (MFI) of CD44 on the surface of CD4^+^ and CD8^+^ T cells, respectively. **(G–L)** Detection of intracellular cytokines after brief *in vitro* restimulation. **(G,J)** IFN-γ, **(H,K)** IL-2, **(I,L)** IL-17; in CD4^+^ and CD8^+^ T cells, respectively. *n* = 5 mice/group. MST, median survival time.

Malt1-ko mice are characterized by the lack of IL-2 production due to impaired downstream TCR signaling (8, 9). These mice were described to have severely reduced frequencies of peripheral Treg, similar proportions of single positive CD4 and CD8 T cells as well as B cells, but with a different activation/memory status in steady-state conditions, as compared to wild-type (Wt) mice (20, 25). As Malt1-deficiency was shown to protect against the development of experimental autoimmune encephalomyelitis (EAE) despite diminished Treg numbers (26, 27), we tested whether it could also protect against graft rejection in a skin transplantation model. B6D2 donor skins were used as allografts for Malt1-ko, control Wt B6 recipients and Wt B6 mice that were treated with CsA (Figure 1A). Graft survival was significantly prolonged in Malt1-ko mice (MST = 25 days, *P* < 0.001) compared to Wt controls (MST = 14 days), while CsA treatment alone had no significant effect on graft survival (MST = 15 days) in this setting (Figure 1B).

Analysis of T-cell responses at the time of rejection in the graft draining lymph nodes (dLN) indicated that, whilst Malt1-deficiency and CsA treatment had no effect on the frequencies of CD4^+^ and CD8^+^ T cells (Figures 1C,D), the activation status (as reflected by the surface expression of CD44) of CD4^+^ but not CD8^+^ T cells was significantly reduced in Malt1-ko mice compared to both untreated control and CsA-treated Wt mice (Figures 1E,F). Malt1-deficiency also significantly impaired the production of IFN-γ and IL-2 effector cytokines by alloreactive CD4^+^ and CD8^+^ T cells (Figures 1G–K). Surprisingly, the frequency of CD4^+^IL-17^+^ T cells was augmented in Malt-ko compared to Wt mice (Figure 1I). Interestingly, while CsA treatment resulted in an expected diminished frequency of alloreactive CD4^+^IL-2^+^ T cells, it had little effect on CD4^+^IFN-γ^+^ and CD8^+^IL-2^+^ T cells and skewed the response toward the expansion of CD8^+^IFN-γ^+^ and IL-17^+^ effector T cells (Figures 1G–L). Taken together, these data indicated that unlike CsA, Malt1-deficiency could effectively inhibit CD4^+^ Th1 and CD8^+^ T-cell alloresponses, thus favoring the prolongation of MHC-mismatched skin graft survival. However, similar to CsA treatment, Malt1-deficiency resulted in the increased generation of Th17 cells that most likely contributed to final, albeit delayed, graft rejection.

### Allograft Rejection in Malt1-ko Mice Correlates With a Lack of Peripheral Treg and Elevated IL-17^+^ Effector T Cells Early After Transplantation

**FIGURE 2:**
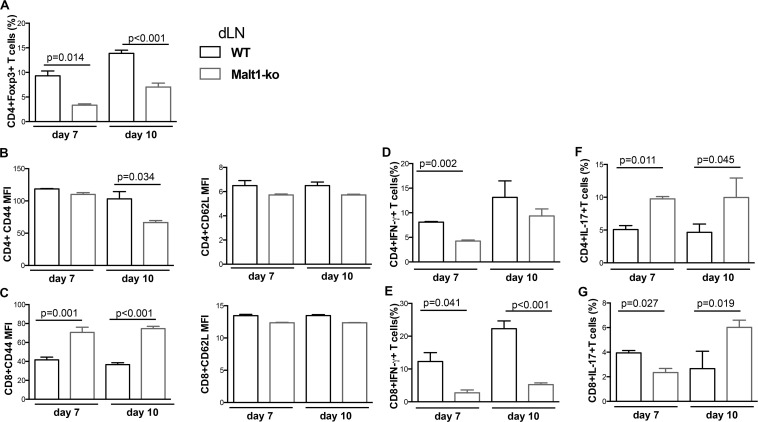
Allograft rejection in Malt1-ko mice correlates with a lack of peripheral Treg and elevated IL-17-producing T cells early after transplantation. Graft draining lymph nodes (dLN) of wild-type (Wt) B6 and Malt1-ko recipients were analyzed by flow cytometry at days 7 and 10 post transplantation of B6D2 skins. Frequency of **(A)** CD4^+^Foxp3^+^ T cells. **(B,C)** Mean fluorescence intensity (MFI) of CD44 and CD62L expression on the surface of CD4^+^ and CD8^+^ T cells, respectively. **(D–G)** Detection of intracellular cytokines after brief *in vitro* restimulation. **(D,E)** IFN-γ, **(F,G)** IL-17; in CD4^+^ and CD8^+^ T cells, respectively. *n* = 5 mice/group.

Although Malt1-ko mice had prolonged graft survival compared to controls, the skin grafts were all, eventually, rejected. To further dissect the mechanisms leading to rejection, we compared T-cell responses in the graft dLN (Figure 2) and spleen (Supplementary Figure 1) of Malt1-ko and Wt recipients of B6D2 skins on days 7 and 10 post transplantation. The frequency of Treg remained lower in Malt1-ko in comparison to Wt mice, even if it tended to increase over time after immune activation (Figure 2A). As compared to their Wt counterparts, CD4^+^ T cells in Malt1-ko mice were less activated by day 10, while paradoxically, CD44 was significantly upregulated on CD8^+^ T cells early after transplantation (Figures 2B,C). Malt1-deficiency impaired the early differentiation of CD4^+^ and CD8^+^ T cells into IFN-γ producing effector cells in response to an allograft (Figures 2D,E), but promoted the differentiation of IL-17-producing effector T cells by day 7 and day 10 for CD4 or CD8 T cells, respectively (Figures 2F,G). Collectively, these data suggested that whilst Malt1-deficiency impaired the differentiation of alloreactive Th1 and CD8^+^IFN-γ^+^ T cells, insufficient peripheral Treg numbers early after transplantation and a skewing toward IL-17-mediated immune responses triggered rejection.

### Malt1-Deficiency Promotes Tolerance to Minor Histocompatibility Antigens-Mismatched Skin Grafts

**FIGURE 3:**
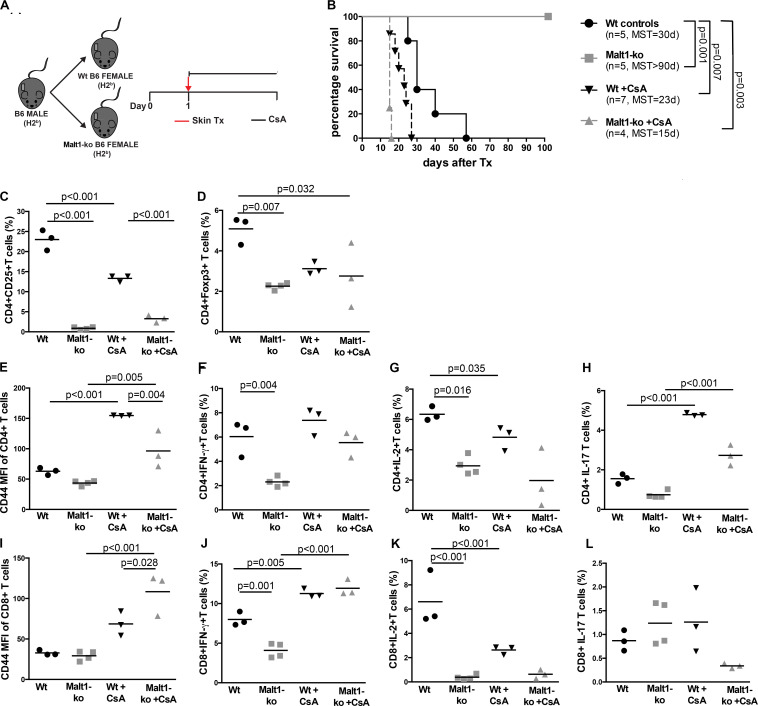
Malt1-deficiency promotes tolerance to minor histocompatibility antigens-mismatched skin grafts. **(A)** Male B6 skins were transplanted to wild-type (Wt) and Malt1-ko female B6 mice that were left untreated or received cyclosporine A (CsA) i.p. daily after transplantation. **(B)** Graft survival. **(C,D)** Frequency of CD4^+^CD25^+^ and CD4^+^Foxp3^+^ T cells, respectively. **(E,I)** Mean fluorescence intensity (MFI) of CD44 on the surface of CD4^+^ and CD8^+^ T cells, respectively. **(F–L)** Detection of intracellular cytokines after brief *in vitro* restimulation. **(F,J)** IFN-γ, **(G,K)** IL-2, **(H,L)** IL-17; in CD4^+^ and CD8^+^ T cells, respectively. *n* = 5 mice/group. MST, median survival time.

**FIGURE 4:**
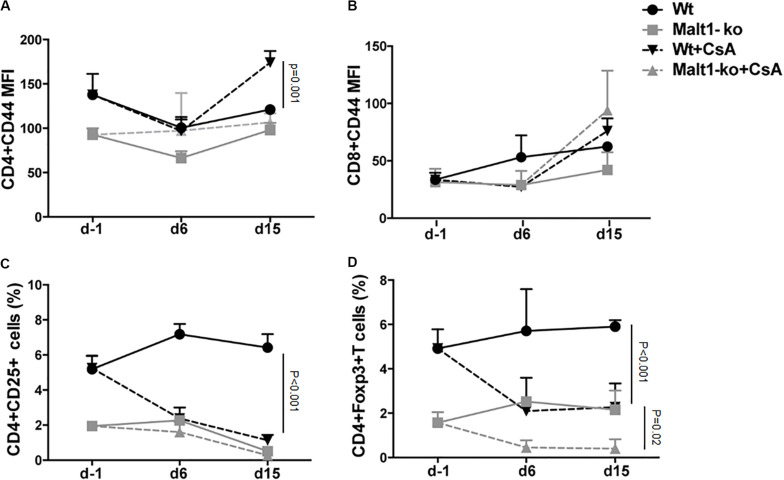
Cyclosporine A treatment gradually decreases peripheral Treg whilst increasing activated effector T cells in Wt and Malt1-ko mice. Male B6 skins were transplanted to wild-type (Wt) and Malt1-ko female B6 mice that were left untreated or received cyclosporine A (CsA) i.p. daily after transplantation. The recipient mice were tail-bled the day before transplantation (d-1), then at day 6 and 15 post transplantation. **(A,B)** Mean fluorescence intensity (MFI) of CD44 expression on the surface of CD4^+^ and CD8^+^ T cells, respectively. **(C,D)** Frequency of CD4^+^CD25^+^ and CD4^+^Foxp3^+^ T cells. *n* = 5 mice/group.

We hypothesized that the immunosuppression provided by Malt1-deficiency may be sufficient to promote graft tolerance in response to a weaker allogeneic stimulation and thus used a MHC-matched but minor-H-mismatched donor-recipient strain combination. Female Malt1-ko, Wt and CsA-treated Wt B6 mice were transplanted with male B6 donor skins (Figure 3A). Female Malt1-ko recipients were tolerant to male donor skins (MST > 100 days) (Figure 3B). Intriguingly, CsA was deleterious in this setting, by accelerating rejection in Wt and breaking tolerance in Malt1-ko recipients, when compared to non-treated Wt mice (MST = 23, 15, and 30 days in Wt + CsA, Malt1-ko + CsA and non-treated Wt, respectively). We next analyzed the peripheral effector T-cell repertoire in the graft dLN at rejection or at day 100 for the tolerant mice. Consistent with their genetic defect, Malt1-ko mice had a reduced population of peripheral Treg compared to Wt rejectors (Figures 3C,D). Exposure to CsA resulted in similar low frequencies of peripheral Treg; however, CD44 was upregulated on CD4^+^ and CD8^+^ T cells (both in Wt and Malt1-ko CsA-treated mice), reflecting the presence of a population of activated T cells that could have promoted acute rejection (Figures 3E,I). Malt1-deficiency was sufficient to prevent the activation and production of pro-inflammatory cytokines by CD4^+^ and CD8^+^ T cells in response to minor-H alloantigens, thus promoting tolerance despite low Treg frequencies. In comparison, CsA treatment also resulted in reduced frequencies of IL-2^+^ effector T cells, but it could not control IFN-γ^+^ effectors or CD4^+^IL-17^+^ T cells (Figures 3F–H,J–L). Therefore, these data indicated that CsA accelerated minor-H-mismatched grafts rejection in both Wt and Malt1-ko mice by augmenting the numbers of effector CD8^+^IFN-γ^+^ and pathogenic CD4^+^IL-17^+^ T cells. These data were further confirmed by analyzing the kinetics of T-cell activation as well as Treg frequencies in the peripheral blood at different time-points after transplantation. In both Wt and Malt1-ko mice, we observed a progressive increase from day 6 to day 15 of the activation marker CD44 on CD4^+^ and CD8^+^ T cells upon CsA treatment, compared to the untreated controls (Figures 4A,B). CsA exposure also strikingly decreased the number of Treg in Wt mice, and to a lower extent, in Malt-ko mice that already had constitutively reduced Treg levels (Figures 4C,D).

### Constitutive Inhibition of Malt1 Protease Activity as in Malt1-ki Mice Allows Only Partial Prolongation of MHC-Mismatched Skin Allograft Survival

**FIGURE 5:**
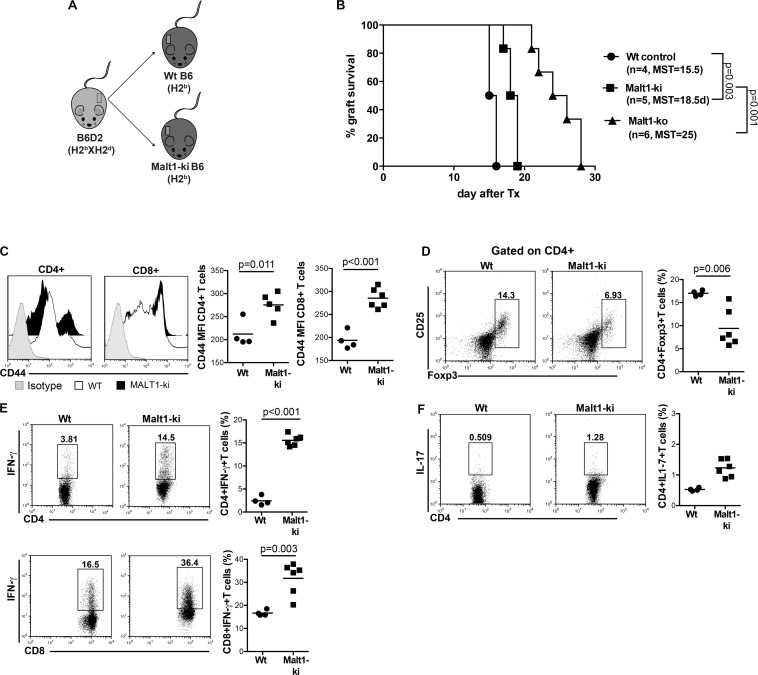
Constitutive inhibition of Malt1 protease activity in Malt1-ki mice only slightly delays graft rejection. **(A)** Recipient Malt1-ki, Malt1-ko and Wt B6 mice received a MHC-mismatched B6D2 skin graft. **(B)** Graft survival. CD4^+^ and CD8^+^ T-cell responses in the graft draining LN (dLN) were analyzed by flow cytometry. **(C)** Mean fluorescence intensity (MFI) of CD44 expression on the surface of CD4^+^ and CD8^+^ T cells. Frequency of **(D)** CD4^+^CD25^+^Foxp3^+^ T cells, **(E)** IFN-γ^+^ CD4^+^ and CD8^+^ T cells, **(F)** IL-17^+^ CD4^+^ T cells. *n* = 4–6 mice/group. MST, median survival time.

We next wanted to evaluate the selective contribution of Malt1 protease activity in controlling alloresponses. To this purpose, we performed transplantation experiments in Malt1-ki mice expressing a catalytically inactive mutant of Malt1 that conserves its scaffold function (Figure 5A). Constitutive inhibition of Malt1 protease activity, as in Malt1-ki B6 recipients, allowed only slight prolongation of the survival of B6D2 skin allografts compared to Wt B6 recipients (MST = 18.5 and 15.5 days in Malt-ki and Wt, respectively. *P* = 0.003) and Malt-ko mice (MST = 25 days) (Figure 5B). This limited effect was consistent with our previous report, describing an intrinsic default in Treg development combined with only partially compromised T-cell activation capacity in Malt-ki mice, resulting in an imbalance between Treg and effector T cells (20). Indeed, in addition to a reduced frequency of Treg, we observed remarkably elevated CD4^+^ and CD8^+^ effector T-cell responses in Malt1-ki at rejection, compared to Wt recipients (Figures 5C–F). Thus, transplantation tolerance was impaired in mice with constitutive inactivation of the enzymatic activity of Malt1.

### Selective Inhibition of Malt1 Protease Activity in Wt Peripheral T Cells Maintains Treg and Attenuates Th17 Differentiation in Response to Alloantigens *in vitro*

**FIGURE 6:**
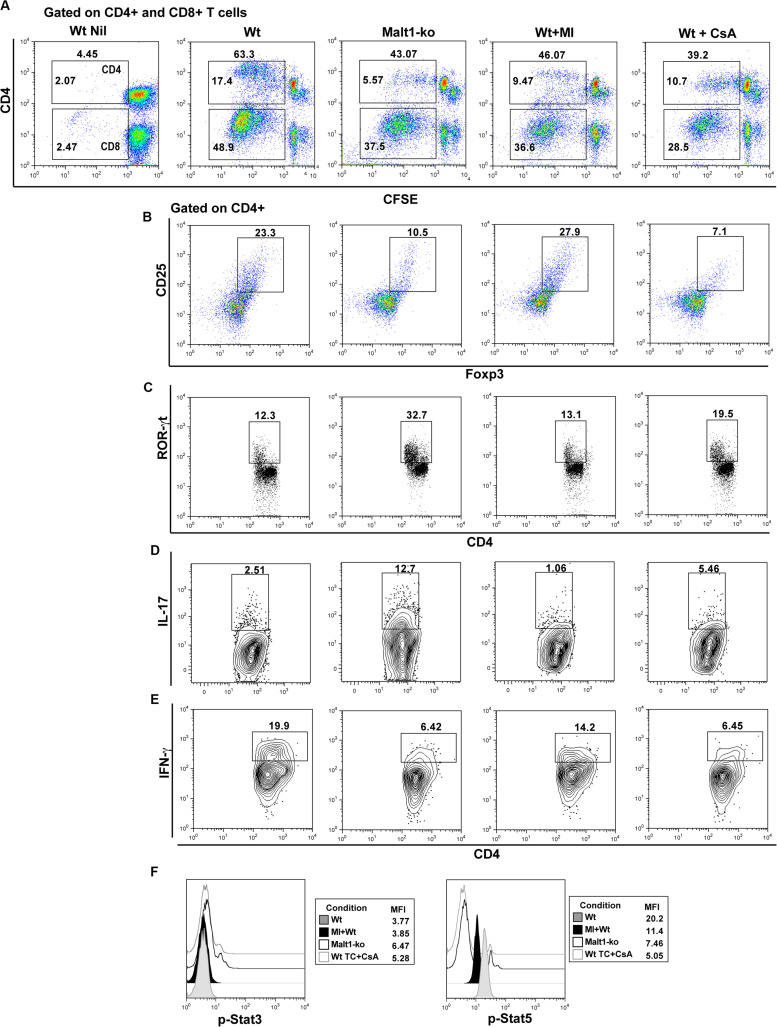
Selective inhibition of Malt1 protease activity in Wt peripheral T cells does not affect Treg but attenuates Th17 differentiation in an alloresponse. Whole T cells (TC) were purified from spleen and lymph nodes of wild-type (Wt) and Malt-ko B6 mice. 1 × 10^5^ CFSE-labeled responder T cells were cultured with 0.2 × 10^5^ allogeneic B6D2 DC for 6 days. Wt T cells cultured alone in medium were used as controls (Wt Nil). As comparison, Wt T cells were treated with either 200 μM Malt1 tetrapeptide inhibitor z-VRPR-fmk (MI), or 100 ng/ml cyclosporine A (CsA). **(A)** CFSE dilutions of dividing CD4^+^ (upper gate) and CD8^+^ (lower gate) T cells on day 6 of culture. Frequency of **(B)** Foxp3^+^CD25^+^, **(C)** ROR-γt^+^, **(D)** IL-17^+^ and **(E)** IFN-y^+^ CD4^+^ T cells (gated on CD4^+^), respectively. **(F)** Phosphorylated (p)-Stat3 and p-Stat5 expression (gated on CD4^+^) analyzed by flow cytometry after short restimulation of T cells with anti-CD3/CD28-coated beads at 37° for 20 min. All culture conditions were performed in triplicate. Flow cytometry dot-plots and histograms data are representative of one out of three independent experiments.

We further investigated whether selectively inhibiting the protease activity of Malt1 in Wt peripheral mature T cells, utilizing the Malt1 tetrapeptide inhibitor z-VRPR-fmk (MI) (23), would result in a better targeting of effector T cells, while preserving peripheral Treg function. Purified whole T cells from B6 Wt and Malt1-ko mice, as well as Wt T cells treated *in vitro* with either MI or CsA, were cultured with allogeneic B6D2 DC and analyzed after a 6-days MLR. Malt1-deficiency, MI and CsA treatments, all significantly decreased the proliferative capacity of CD4^+^ and CD8^+^ T cells *in vitro* in response to alloantigens (Figure 6). Complete Malt1-deficiency most effectively inhibited the proliferation of CD4^+^ T cells compared to MI and CsA, while CsA was more effective on the proliferation of CD8^+^ T cells (Figure 6A). CsA treatment resulted in strongly reduced proportions of CD4^+^CD25^+^Foxp3^+^ T cells at the end of the co-culture. In contrast, MI treatment did not significantly affect the *in vitro* expansion of Treg in the MLR, compared to untreated Wt T cells (Figure 6B). Similar to the *in vivo* data, Malt1-deficiency impaired the differentiation of CD4^+^IFN-γ^+^ Th1 cells when compared to control Wt T cells, while the expression of the Th17 transcription factor ROR-γt and the frequency of CD4^+^IL-17^+^ T cells were dramatically elevated. MI treatment of Wt T cells less effectively inhibited Th1 cells, but at the same time did not increase Th17 differentiation (Figures 6C–E). Overall, in contrast to full Malt1-deficiency or CsA treatment, selective inhibition of Malt1 protease activity in peripheral Wt T cells maintained Treg whilst attenuating Th17 differentiation *in vitro*.

We next assessed the effects of MI treatment on TCR downstream IL-2-dependent signaling, as it critically influences the differentiation of T helper subsets and is required for the maintenance and function of Treg (28, 29). We thus analyzed signal transducer and activator of transcription (Stat)3 and Stat5 activation after brief *in vitro* stimulation of T cells. Our data showed that Malt1-ko and CsA-treated T cells, both of which promoted the differentiation of Th17 cells, had slightly increased phosphorylated (p)-Stat3 but significant low p-Stat5 levels (Figure 6F). In comparison, Wt and MI-treated T cells in which the subset of CD4+Foxp3+ T cells was maintained in the alloMLR, had a higher expression of p-Stat5. Collectively, these data were consistent with the fact that selective inhibition of Malt1 protease activity in mature T cells, in contrast to Malt1-deficiency or CsA treatment, maintains Treg frequencies while attenuating Th17 differentiation.

### The Malt1 Protease Inhibitor Mepazine Regulates Human T Cells Alloresponse *in vitro* and Prolongs MHC-Mismatched Skin Allograft Survival in Wild-Type Mice

**FIGURE 7:**
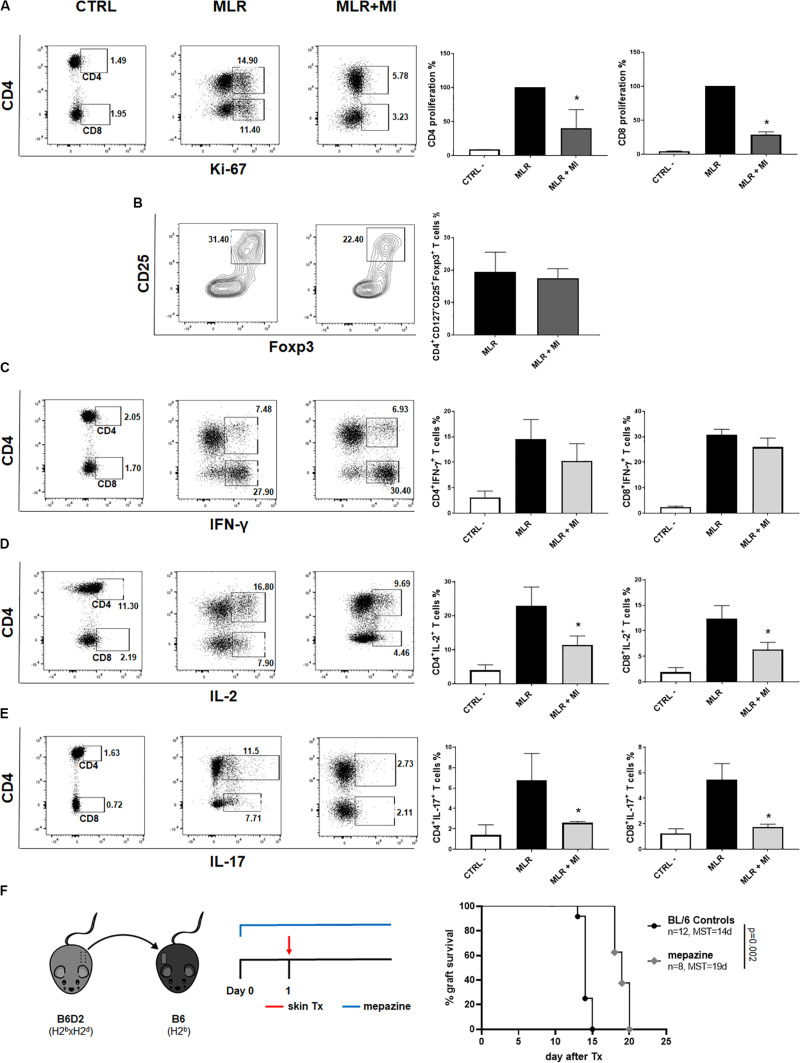
The Malt1 protease inhibitor mepazine regulates human T cells alloresponse *in vitro* and prolongs MHC-mismatched skin allograft survival in wild-type mice. PBMC from healthy donors were cultured alone (CTRL, left dot-plot panels) or co-cultured with irradiated allogeneic APC in a MLR, without or with the Malt1 inhibitor mepazine (MI, 3 μg/ml) (MLR and MLR + MI, middle panel and right panel dot-plots, respectively). Cells were harvested on day 7 of co-culture and analyzed by flow cytometry **(A)** CD4 (upper gate) and CD8 (lower gate) T cells proliferation was assessed by Ki-67 staining. Frequency of **(B)** CD4^+^CD25^+^CD127^–^ Foxp3^+^ T cells, **(C)** IFN-y^+^, **(D)** IL-2^+^ and **(E)** IL-17^+^ CD4^+^ (upper gate) and CD8^+^ (lower gate) T cells, respectively. All culture conditions were performed in triplicates. Flow cytometry data are representative of one out of three independent experiments. Means with SD, *P < 0.05. **(F)** Recipient wild-type (Wt) B6 mice received a MHC-mismatched B6D2 skin allograft, and were left untreated or received mepazine i.p. daily, starting the day before transplantation. Graft survival was monitored daily. MST, median survival time.

Next, we wanted to evaluate the potential of selective targeting of Malt1 protease activity in clinical transplantation. We tested the effect of mepazine hydrochloride, a clinically approved drug for psychological disorders, which was later shown to be a potent cell-permeable inhibitor of Malt1 protease (30). PBMC isolated from healthy volunteers were cultured for 7 days in the presence of allogeneic irradiated APC, without or with the addition of mepazine. Mepazine treatment significantly decreased the proliferation of human CD4^+^ and CD8^+^ T cells (Figure 7A) in the alloMLR. Importantly, the inhibition of Malt1 by mepazine did not affect the frequency of the CD4^+^CD25^+^ CD127^–^Foxp3^+^ T cell population at the end of the co-culture (Figure 7B). While the drug did not significantly reduce the production of IFN-γ in both CD4^+^ and CD8^+^ T cells in our culture conditions (Figure 7C), we observed a significant decrease in the frequency of IL-2^+^ CD4^+^ and CD8^+^ T cells (Figure 7D) and CD4^+^IL-17^+^ T cells (Figure 7E). Therefore, selective targeting of Malt1 protease in human PBMC sustains Foxp3^+^ Treg cells and impairs Th17 differentiation during alloresponses *in vitro*. Finally, when used *in vivo* in our transplantation model (Figure 7F), mepazine prolonged allograft survival in Wt B6 recipients of B6D2 skin grafts (MST = 19 and 14.5 days in mepazine-treated and non-treated, respectively, *P* = 0.002). The prolongation of graft survival was significant when compared to the use of CsA (MST = 15 days, Figure 1B) in the same experimental setting and interestingly similar to the graft survival obtained in Malt-ki B6 mice (MST = 18.5 days, Figure 5B).

## Discussion

Immunotherapeutic strategies targeting alloantigen-activated T cells remain the cornerstone of current immunosuppressive management after SOT (3) and our study analyzed for the first time the role of Malt1-dependent TCR downstream signaling in an experimental model of allogeneic transplantation. Consistent with studies in other models (8, 9, 20, 27), Malt1-deficiency attenuated alloreactive Th1 responses resulting in prolonged survival of MHC-mismatched skin allografts, despite very low frequencies of peripheral Treg in these mice (25). However, tolerance was not achieved in our experimental setting and we observed a progressive increase in IL-17-producing T cells after transplantation and at rejection. Since Malt1-ko mice have impaired B-cell development and reduced antibody production (8, 9), we propose that Th17 cells played a key role in mediating late rejection. In the same experimental setting, mice treated with the CNI CsA were not protected and rejected their grafts with similar kinetics as Wt recipients. As compared to Malt1-ko, IFN-γ^+^ and IL-17^+^ effector T cells were increased at rejection in CsA-treated recipients. Thus, our data suggest the superiority of targeting Malt1 in preventing the activation of alloreactive T cells and acute rejection of MHC-mismatched grafts.

Graft rejection has been associated with a predominant Th1 response. It was previously described that in the absence of a functional Th1 pathway, as in T-bet^–/–^ mice, Th17 cells could cause allograft rejection (2). In clinical studies of kidney and lung transplantation, elevated levels of IL-17 mRNA or protein were associated with accelerated graft rejection (31). In line with these data, we report that while impeding Th1 activation and IL-2 production, CsA treatment and Malt1-deficiency resulted in elevated IL-17^+^ T cells after allogeneic skin transplantation. The elevated IL-17 response observed in Malt1-ko mice after transplantation is in contrast to some reports that described the requirement of Malt1, and in particular of its protease activity, for Th17 differentiation (12, 13). Because TCR-downstream canonical NF-κB activation is blunted by Malt1-deficiency, our findings suggest that in some settings, such as during an alloresponse and in an inflammatory microenvironment, Th17 differentiation could be promoted in Malt1-ko mice via an alternative T-cell signaling pathway. Indeed, the ERK-MAPK pathway was reported to contribute to the regulation of the reciprocal differentiation between Treg and Th17 cells in autoimmunity models such as EAE and colitis (32). Whether the ERK-MAPK or other non-canonical NF-κB signaling pathways were up-regulated and played a role in increased Th17 differentiation in our transplantation model remains to be investigated (32, 33). Finally, we observed increased CD8^+^IL-17^+^ T cells (Tc17) in Malt1-ko and CsA-treated mice after allogeneic transplantation. Although unconventional in the alloresponse, Tc17 have been reported to contribute together with CD4^+^IL-17^+^ T cells to the induction of EAE in the absence of Treg (34). Taken together, our results suggest that Malt1-deficiency and CsA treatment both resulted in the reduced frequency of peripheral Treg and impaired Th1 responses, but promoted the differentiation of Th17 cells that mediated allograft rejection.

In an experimental transgenic mouse model targeting the CBM complex of NF-κB activation, it was shown that whilst defective NF-κB intrinsic signaling in T cells promoted long-term cardiac allograft survival, more immunogenic primary skin allografts where rejected (17). We therefore investigated whether immunosuppression provided by Malt1-deficiency was sufficient to promote graft tolerance in a weaker alloresponse. We found that Malt1-deficiency induced robust tolerance to minor-H-mismatched skin grafts and, even with low peripheral Treg numbers, was sufficient to reduce the activation and pro-inflammatory cytokines production of alloreactive CD4^+^ and CD8^+^ T cells. CsA treatment however, significantly reduced graft survival of both Wt and Malt1-ko mice in this donor-recipient strain combination (35, 36), further highlighting different mechanisms of regulation of TCR downstream signaling by calcineurin and Malt1. We observed that CsA treatment progressively decreased the frequency of peripheral Treg in both Wt and Malt1-ko mice. Furthermore, as opposed to Malt1-deficiency, CsA treatment could not efficiently control the activation of alloreactive CD4^+^ and CD8^+^ T cells, resulting in a skewed response toward effector CD8^+^IFN-γ^+^ and pathogenic CD4^+^IL-17^+^ T cells in the absence of IL-2 signaling. The combined inhibition of NFAT and NF-κB signaling by treating Malt1-ko mice with CsA further decreased Treg numbers and augmented effector responses resulting in accelerated allograft rejection. Taken together, these results indicated that targeting Malt1 offered some advantages over the use of CsA as immunosuppressive strategy after transplantation, but also indicates that combined treatment offers no additional benefit.

Following on our results in Malt1-ko mice, we investigated how selectively targeting the protease activity of Malt1 would affect alloresponses. As previously reported, we confirmed excessive immune activation in Malt1-ki adult mice in response to an allograft, associated with an intrinsic T-cell developmental defect (including thymus-derived Treg) and dysregulated immune balance between Treg and effector T cells (20, 25, 37–40). Because of the known *in vivo* toxicity of the fmk component of the Malt1 protease selective inhibitor z-VRPR-fmk (MI), we used an *in vitro* model to test the effect of MI on mature peripheral T cells (23). In comparison to complete Malt1-deficiency and CsA treatment, MI treatment of Wt peripheral mouse T cells attenuated T-cell proliferation in response to alloantigens, while the subset of preexisting Treg was not affected and Th17 responses were not promoted. Stat3 and Stat5 activation were reported to play a key role in the reciprocal balance between Treg and Th17 cells. Indeed, TCR-induced IL-2-mediated activation of Stat5 promotes the maintenance and suppressive function of Foxp3^+^ Treg and limits the responsiveness of T cells to IL-6 (28, 41, 42). Activated p-Stat5 also competes with p-Stat3 for binding to the *IL-17* gene locus, preventing Th17 cell differentiation (28, 29, 43). Thus, the inhibition of IL-2 signaling (and Stat5) in Malt1-ko and after CsA treatment would promote Stat3 usage and shift the cells toward Th17 differentiation. Accordingly, flow cytometry analysis revealed elevated Th17 responses in Malt1-ko and CsA-treated alloreactive T cells, which corresponded to increased p-Stat3 and decreased p-Stat5 expression in comparison to Wt and MI-treated Wt T cells. These data further supported our *in vivo* findings that had shown enhanced Th17 responses in Malt1-ko and CsA-treated transplant recipients.

Finally, in the perspective of clinical application, we explored the effect of selective targeting of Malt1 protease activity in regulating human PBMC alloresponses. We chose to use mepazine, a drug already available in the clinic and with a known safety profile. Besides their indication as antipsychotic drugs, phenothiazine derivatives such as mepazine were shown to be potent selective small molecule inhibitors of Malt1 protease (44, 45). Mepazine was successfully tested as a therapy in Malt1-dependent tumor models (21, 30, 46) as well as in EAE (27, 47). In our experimental *in vitro* setting using human PBMC, mepazine treatment attenuated T-cell proliferation in response to alloantigens in 7-days MLRs, without promoting Th17 differentiation. Importantly, preexisting Treg were not negatively affected despite decreased frequencies of  IL-2^+^ effector T cells. Based on these encouraging *in vitro* data, we explored the effect of mepazine treatment in our *in vivo* transplantation model. In initial experiments, mepazine slightly improved MHC-mismatched allograft survival in Wt B6 recipients, in a setting where CsA treatment has no beneficial effect. Interestingly, the effect was similar to the extent of graft survival prolongation observed in Malt-ki mice. Further studies are however needed to confirm the therapeutic potential of mepazine and other available selective Malt1 protease inhibitors in transplantation. First, we have to define the optimal dose and duration of mepazine treatment in our transplantation model. Second, the effect of mepazine may be optimized if combined with other immunomodulatory drugs, in order to control the clonal expansion of alloreactive effector T cells and preserve (or expand) the pool of allospecific Treg, as suggested by our previous experimental data (5, 48).

Besides mepazine, potent selective Malt1 paracaspase pharmacologic inhibitors have been described that may also be suited in the setting of solid organ transplantation (44, 49–51). The efficacy/safety profile of these compounds need to be tested in our experimental model, in particular regarding the effect of prolonged use on the pool of effector and regulatory immune cell subsets. Indeed, unless tolerance to alloantigens can be induced, life-long immunosuppressive therapy is required after SOT. In this regard, a recent preclinical study has revealed potential safety issues regarding prolonged *in vivo* Malt1 protease inhibition, in particular regarding the deleterious effect on peripheral Treg (51). In the study, the authors show that as opposed to mepazine, the chronic administration of a novel potent inhibitor induced a dose-dependent reduction in Treg with associated autoimmunity, reminiscent of the Malt-ki mice. Therefore, less potent Malt1 protease inhibition as achieved with mepazine, or less selective Malt1 inhibition may prove to be better strategies to preserve Treg homeostasis in autoimmune diseases and after SOT. Finally, data suggest Malt1-independent therapeutic effects of mepazine in inflammatory disease models that may also prove beneficial in transplantation (52).

In summary, our study highlights the potential but also the possible limitations of targeting Malt1 (either scaffold or protease) in allogeneic responses. We propose that, in the context of solid organ transplantation, targeting Malt1 signaling may prove as an advantageous alternative to CNI in immunosuppressive protocols. Furthermore, combination therapies including the administration of a selective inhibitor of Malt1 protease activity might offer a better control of alloreactive Th1 responses while preserving preexisting Treg in the peripheral T-cell pool, and without promoting Th17 differentiation. Therefore, besides current developments in the field of oncology, there is the need for research and validation in solid organ transplantation of selective Malt1 inhibitors that can be safely used as part of immunosuppressive protocols in the clinic.

## Data Availability Statement

The raw data supporting the conclusions of this article will be made available by the authors, without undue reservation.

## Ethics Statement

The studies involving human participants were reviewed and approved by Commission cantonale d’éthique de la recherche sur l’être humain (CER-VD). The participants provided their written informed consent to participate in this study. The animal study was reviewed and approved by Canton de Vaud veterinary authorization.

## Author Contributions

LG and DG designed and performed the research, analyzed and interpreted the data, and wrote the manuscript. JM and J-CW performed the research. JM, OG, and MT interpreted the data and wrote the manuscript. All authors contributed to the article and approved the submitted version.

## Conflict of Interest

The authors declare that the research was conducted in the absence of any commercial or financial relationships that could be construed as a potential conflict of interest.
