# Supplementary material for: Therapeutic Potential of Targeting Malt1-Dependent TCR Downstream Signaling to Promote the Survival of MHC-Mismatched Allografts
Source: Front Immunol. 2020 Sep 11;11:576651. doi: 10.3389/fimmu.2020.576651 (PMC7517581; doi:10.3389/fimmu.2020.576651)
Supplement: Supplementary file 1 [file Table_1.DOCX]

Supplementary Material

**Therapeutic potential of targeting Malt1-dependent TCR downstream signaling to promote the survival of MHC-mismatched allografts**

**Authors:**

**Lerisa Govender, Josip Mikulic, Jean-Christophe Wyss, Olivier Gaide, Margot Thome, Dela Golshayan^*^**

*** Correspondence:** Prof D Golshayan: [Dela.Golshayan@chuv.ch](mailto:Dela.Golshayan@chuv.ch)

## Supplementary Figures


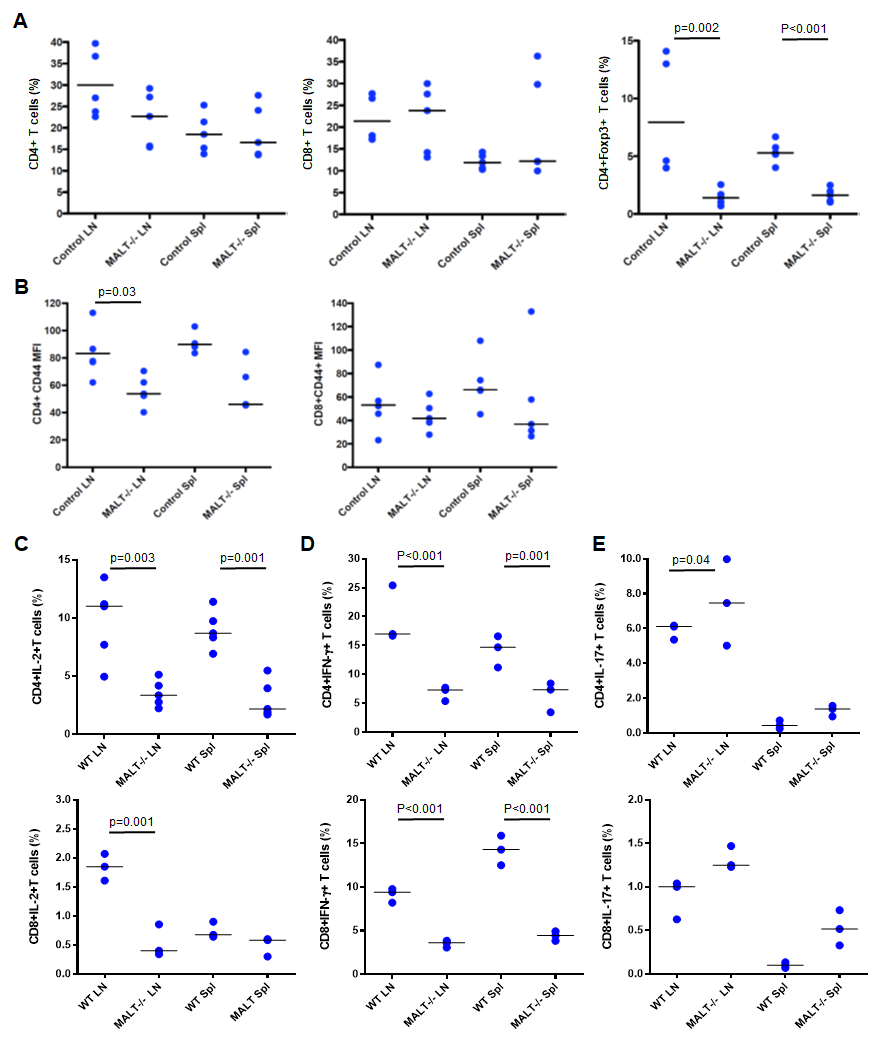


**Supplementary Figure 1. Allograft rejection in Malt1-ko mice correlates with a lack of peripheral Treg and elevated IL-17 production early after transplantation**. Graft draining lymph nodes (LN) and spleens (Spl) of wild-type (control, WT) B6 and Malt1-ko (MALT-/-) recipient mice were analyzed by flow cytometry at day 10 post transplantation of B6D2 skins. Frequency of **A.** CD4^+^, CD8^+^ and CD4^+^Foxp3^+^ T cells. **B.** Mean fluorescence intensity (MFI) of CD44 expression on the surface of CD4^+^ and CD8^+^ T cells, respectively. **C-E.** Detection of intracellular cytokines after brief *in vitro* restimulation. **C.** IL2, **D.** IFN-γ, **E.** IL-17; in CD4^+^ and CD8^+^ T cells, respectively. n=3-5 mice/group.
